# Cardiorespiratory alterations in rodents experimentally envenomed with *Hadruroides lunatus* scorpion venom

**DOI:** 10.1186/s40409-016-0076-5

**Published:** 2016-07-15

**Authors:** Fernanda Costal-Oliveira, Clara Guerra-Duarte, Maira Souza Oliveira, Karen Larissa Pereira de Castro, Leticia Lopes-de-Sousa, Aline Lara, Enéas Ricardo de Morais Gomes, Cesar Bonilla, Sílvia Guatimosim, Marília Martins Melo, Carlos Chávez-Olórtegui

**Affiliations:** Department of Biochemistry and Immunology, Institute of Biological Sciences, Federal University of Minas Gerais, Belo Horizonte, CP: 486 CEP: 31270-901 MG Brazil; College of Veterinary Medicine, Federal University of Minas Gerais, Belo Horizonte, MG Brazil; Department of Physiology and Biophysics, Institute of Biological Sciences, Federal University of Minas Gerais, Belo Horizonte, MG Brazil; Instituto Nacional de Salud, Universidad Nacional Mayor de San Marcos y Universidad Científica del Sur, Lima, Peru

**Keywords:** *Hadruroides lunatus* venom, Cardiorespiratory alterations, Electrocardiography, Immunofluorescence, Calcium transient

## Abstract

**Background:**

*Hadruroides lunatus* is the most abundant scorpion species in the Peruvian central coast, where most of the accidents involving humans are registered. In spite of its prevalence, there are only very few studies on *H. lunatus* envenomation. The aim of the present study was to analyze the cardiorespiratory alterations caused by *H. lunatus* envenomation in rodents.

**Methods:**

Wistar rats injected with *H. lunatus* scorpion venom were submitted to electrocardiography. After euthanasia, rat lungs were collected and histopathologically analyzed. Mouse cardiomyocytes were used to perform immunofluorescence and calcium transient assays. Data were analyzed by ANOVA or Student’s *t*-test. The significance level was set at *p* < 0.05.

**Results:**

It was observed that *H. lunatus* venom increased heart rate and caused arrhythmia, thereby impairing the heart functioning. Lungs of envenomed animals showed significant alterations, such as diffuse hemorrhage. In addition, immunofluorescence showed that *H. lunatus* venom was capable of binding to cardiomyocytes. Furthermore, mouse ventricular cardiomyocytes incubated with *H. lunatus* venom showed a significant decrease in calcium transient, confirming that *H. lunatus* venom exerts a toxic effect on heart.

**Conclusion:**

Our results showed that *H. lunatus* venom is capable of inducing cardiorespiratory alterations, a typical systemic effect of scorpionism, stressing the importance of medical monitoring in envenomation cases.

## Background

**Fig. 1:**
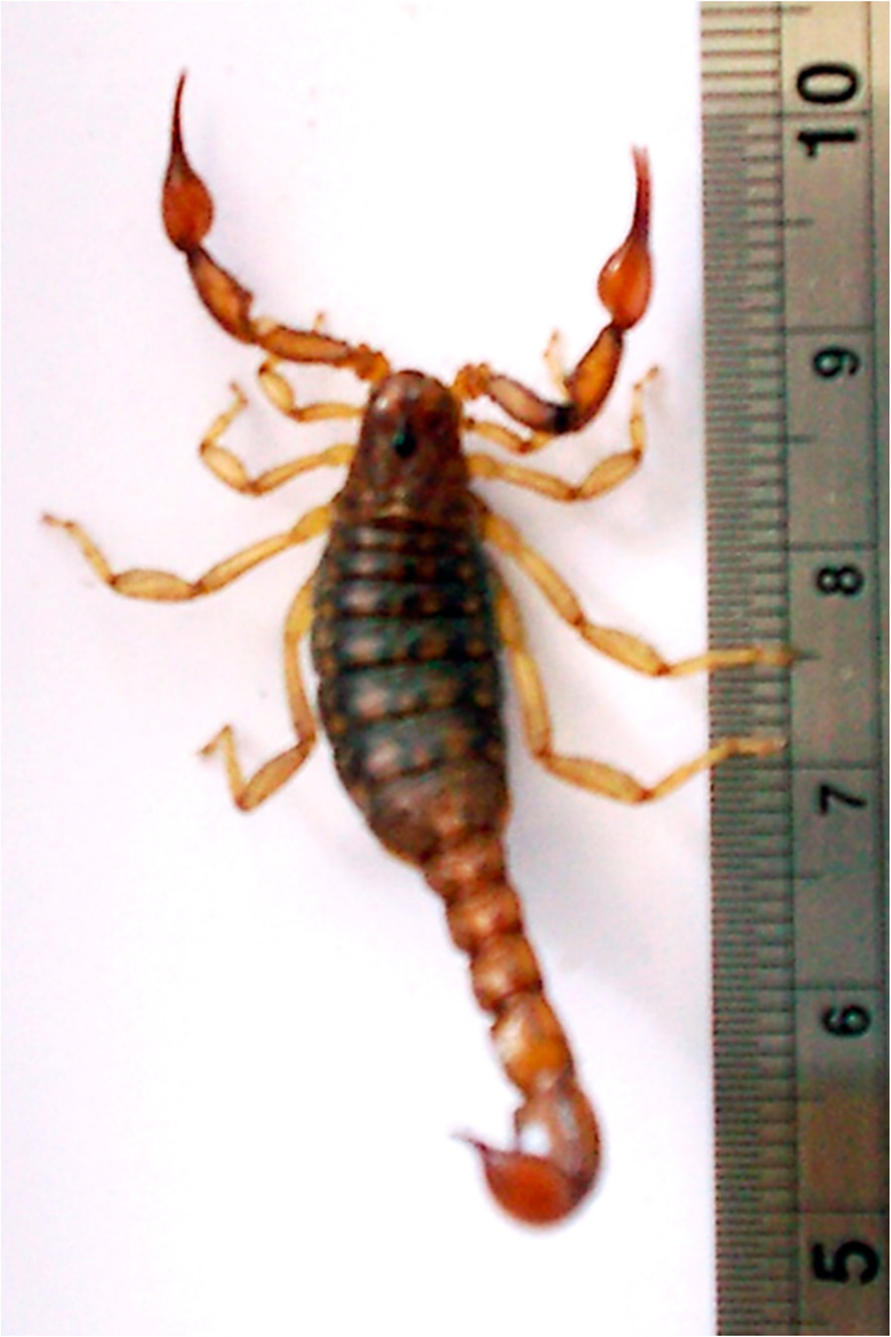
Photo of a specimen of *Hadruroides lunatus* collected in Lima, Peru. This scorpion presents brown coloration and about 5 cm long

The genus *Hadruroides* comprises 22 species of scorpions, distributed throughout Ecuador, Peru, northern Chile and islands around Galapagos, occupying habitats predominantly of arid climate [1]. Scorpions from the species *Hadruroides lunatus* are abundant in Peruvian central coast, particularly around the city of Lima, in rocky areas with “lomas” formations. This species comprises small to medium-sized scorpions that have brownish coloration, with dorsal lighter spots (Fig. 1). *H. lunatus* differs from other *Hadruroides* scorpions by the curved morphology of the pedipalp fixed finger, creating a gap when fingers are closed, and by the rectangular shape of the spots on the tergites [2].

*H. lunatus* is the most medically relevant species in Peru [3]. During 2009, the Health Ministry of Peru [4] reported 41 cases of human accidents caused by this scoprion in Lima. Although these stings are not considered lethal, intense pain, edema, ulceration and necrosis are among the reported symptoms and signs. *H. lunatus* venom has not been extensively studied and there are not sufficient case reports describing human envenomation by this species. However, considering our previous works [5, 6], we hypothesize that this scorpion has potential to cause significant damage to their victims.

In a first attempt to characterize the effects of this venom, our group reported initial data of *H. lunatus* experimental envenomation in rodents [5, 6]. Although *H. lunatus* scorpion venom (*Hlsv*) was classified as moderately toxic when compared with *Tityus* spp. venoms, symptoms such as excitability, agitation, salivation, eye secretions, convulsions, leg paralysis, as well as serological, biochemical and enzymatic alterations were detected in envenomed animals. These symptoms closely resemble those produced by the venom of scorpions pertaining to the Buthidae family, which contains the most medically relevant species that possess, in some cases, neurotoxins in their venoms [7, 8]. These molecules are peptides that act on ion channels and result in great release of neurotransmitters, seriously affecting hemodynamic and cardiorespiratory systems [9, 10].

These previous works suggested *Hlsv* may have cardiotoxic effects, since its activity was associated with high serum levels of creatine kinase (CK) and its isoenzyme MB (CK–MB) [5]. The aim of this study was to confirm this possible cardiotoxic activity of *Hlsv*.

## Methods

### Animals and venom

Twelve male Wistar rats (weighing 100–150 g) and four male C57BL/6 (18–22 g) mice were maintained at the animal facility of the Institute of Biological Sciences, Federal University of Minas Gerais (UFMG), Belo Horizonte, MG, Brazil, and received water and food under controlled environmental conditions. The experimental protocols were approved by the Ethics Committee on the Use of Laboratory Animals of UFMG (CETEA-UFMG protocol 092/11).

*H. lunatus* scorpions were collected in the region of Atocongo (Lima, Peru) and maintained in the National Institute of Health (INS), in Lima, Peru. Scorpions were kept in plastic boxes with water *ad libitum* and fed weekly with cockroaches. Venom was obtained by telson electrical stimulation (12 V) [11]. The venom was diluted in Milli-Q water and stored at −20 °C until use. Protein concentration was measured by the Lowry method [12].

### SDS-PAGE

Different amounts (5, 10 and 20 μg) of Hlsv were diluted in sample buffer under reducing conditions and separated in 15 % SDS-PAGE gel, according to Laemmli [13]. The gel ran at 200 V and was stained with silver.

### Electrocardiography (ECG)

Rats were anaesthetized using 2.5 % isoflurane with a Metalvet Plus anesthetic inhaler (Metalvet, Brazil) and placed in supine position. Pre-anesthetic medication (morphine 2.5 mg/kg and diazepam 2.5 mg/kg) was administrated via intramuscular injections [14]. Electrodes were attached to forelimbs and hindlimbs. Control group (*n* = 6) received 0.4 mL of Milli-Q water via subcutaneous (SC) injection, whilst Hlsv treated group (*n* = 6) received Milli-Q water containing 750 μg of Hlsv. This dose was chosen as the amount of venom corresponding to one third of the LD_50_ stablished for this venom [5]. Computer ECG (ECG-PC TEB, Brazil) tracings were taken prior to the experiment (T0) and variables were analyzed each 5 min throughout the examination, comprising seven time points (T0, T5, T10, T15, T20, T25, T30). Heart rate (HR), heart rhythm, wave measurement and intervals were evaluated. As the ECG software gives the RR interval in millisecond (ms), the heart rate (beats per minute) was calculated by dividing 60,000/RR interval (ms), as 1 min corresponds to 60,000 milliseconds. ECG was recorded at speed of 50 mm/s, sensitivity of 2 N and lead II was considered for analysis.

### Histopathological examination

Rats were euthanized by hypovolemia under anesthesia and submitted to necropsy. Lungs were removed, fixed in 10 % buffered formalin and embedded in paraffin [15]. Histological sections (4 mm) were stained with hematoxylin and eosin, and analyzed in optical microscope.

### Immunofluorescence and calcium transient measurements

Cardiac ventricular myocytes were isolated from C57BL/6 mice by standard enzymatic solution, as previously described [16]. Briefly, animals were euthanized; hearts quickly removed and perfused using a customized Langendorff apparatus with a solution containing type II collagenase (Worthington, USA). After isolation, cells were maintained in Dulbecco’s modified Eagle’s medium (DMEM – Sigma, USA) containing 10 % of fetal bovine serum until use.

For immunofluorescence assay, 500 μL of a solution containing freshly isolated cardiomyocytes (in DMEM containing 10 % fetal bovine serum) was incubated with 0.05 or 2 μg/mL of *Hlsv* at room temperature under agitation for 30 min, followed by incubation with 200 μg/mL of rabbit IgG anti-*Hlsv* for 30 min. Cells were centrifuged, the supernatant removed, and cell pellet was resuspended in medium containing anti-rabbit IgG conjugated to Alexa Fluor 488 (Invitrogen, USA) for 30 min. After centrifugation, the supernatant was removed and 500 μL of fresh medium was added. Images were acquired with a Zeiss LSM 510META confocal microscope (Zeiss Jena, Germany) and analyzed with ImageJ software (NIH, USA). Control cells were incubated only with anti-rabbit IgG conjugated to Alexa Fluor 488 or with anti-*Hlsv* IgG plus IgG conjugated to Alexa Fluor 488.

To measure the intracellular calcium (Ca^2+^) transient, cardiomyocytes were incubated with a calcium sensitive fluorescent probe (fluo 4 AM – 5 μmol/L) for 30 min, and then with 0.05 μg/mL of *Hlsv* for 5–20 min. Calcium transient amplitude was examined in field-stimulated cells at 1 Hz, with a square pulse of 5 ms and 30 V. After application of eight electric pulses, a line-scan imaging was performed in the longitudinal axis of the cells with an acquisition frequency of 1.54 ms, using Zeiss 510 Meta confocal microscope. Thirty-four cells were analyzed in the control group and 32 in *Hlsv* group.

### Statistical analysis

All variables were submitted to normality and homoscedasticity analyses and then analysis of variance (ANOVA). Parametric variables were studied by Student-Newman-Keuls (SNK) posttest and non-parametric variables were evaluated by either Kruskal-Wallis or Friedman posttests. Regression analysis was accessed for HR throughout all time points. Significance was considered for 5 % (*p* < 0.05). Analyses were done in R (2.11 version) software program. Student’s *t*-test was used to access significance of variability among groups in calcium transient analysis.

## Results

### SDS-PAGE

**Fig. 2:**
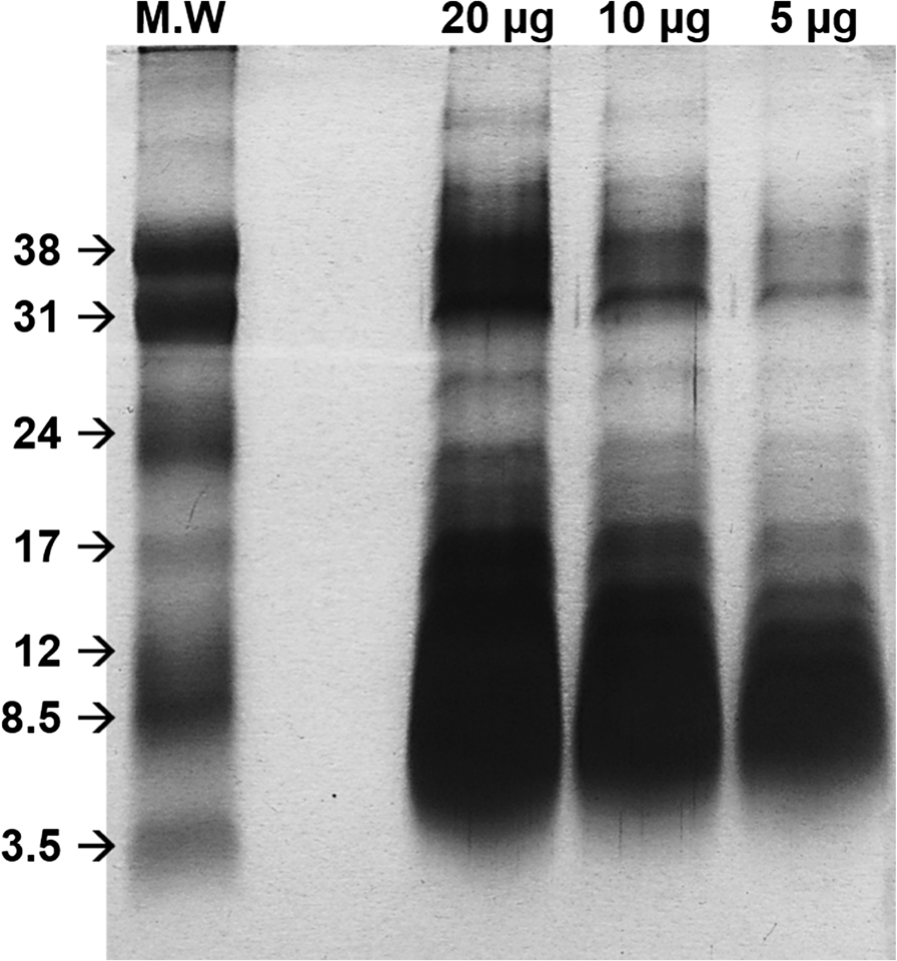
SDS-PAGE 15 % of *Hlsv.* In lane 1, the low molecular weight marker. In the other lanes, 20, 10 and 5 μg of *Hlsv* under reducing conditions. The gel was silver-stained

To evaluate venom content, electrophoresis of different amounts of *Hlsv* (5, 10 and 20 μg) under reducing conditions was performed. The venom profile showed, in addition to a high content of low molecular weight compounds compatible with neurotoxins, a considerable amount of proteins in the range between 12 and 14 kDa. Another group of proteins were also visualized above 30 kDa (Fig. 2).

### Electrocardiographic analysis

At T0, all animals from both groups showed similar ECG tracings with no visible alterations. As usual, RR interval distance was considered to calculate the HR (Table 1). The mean HR on *Hlsv* group significantly increased from T0 to T5. The RR interval from an animal from *Hlsv* group went from 133 ms (HR of 451 bpm) at T0 to 97 ms (HR of 619 bpm) at T5. It means that after 5 min of envenomation, animals presented early signs of poisoning, demonstrated by the significant increase of HR (mean of 424 to 463 bpm), being detected a HR of 619 bpm in one of the animals. At this time (T5), no difference was observed on the control group. At T10, the HR of *Hlsv* group decreased to a value similar to T0, remaining normal up to T30. Within *Hlsv* group, HR values fitted cubic regression (*R*^2^ = 74.36 %; *p* = 0.0486) and T5 indicated the highest HR value (*p* = 0.0380), being others similar to T0 (Table 1). Within control group, HR values fitted simple linear regression (*R*^2^ = 93.04 %; *p* = 0.0004) and no differences were detected in all times.

**Table 1:** Heart rate and T wave amplitude alterations

Time (T)	Heart rate (bpm)		T wave amplitude (mV)	
min	Control	*Hlsv*	Control	*Hlsv*
T0	398.1 ± 25.1	420.04 ± 36.9	0.07 ± 0.03	0.07 ± 0.03
T5	**406.6 ± 22.1**	**463.37 ± 40.9** ^**ab**^	0.08 ± 0.04	0.09 ± 0.04
T10	420.1 ± 20.0	419.02 ± 29.3	**0.08 ± 0.04**	**0.14 ± 0.03** ^**cd**^
T15	426.6 ± 22.1	416.23 ± 24.0	**0.08 ± 0.03**	**0.15 ± 0.02** ^**cd**^
T20	443.3 ± 13.7	402.30 ± 17.4	**0.07 ± 0.03**	**0.17 ± 0.03** ^**cd**^
T25	450.0 ± 15.2	397.56 ± 20.8	**0.06 ± 0.03**	**0.19 ± 0.02** ^**cde**^
T30	446.6 ± 18.8	410.43 ± 37.5	**0.06 ± 0.02**	**0.19 ± 0.03** ^**cde**^

*Hlsv* group presented significant increase of heart rate at T5 (highlighted in bold). ^a^vs. control group T5; T5 ^b^vs. *Hlsv* in all other time points. *Hlsv* group presented increased T waves at all times after T10 (highlighted in bold). ^c^vs. control group at the same time point; ^d^T10 vs. venom group at T0 and T5; ^e^T25 and T30 vs. venom group at T10 and T15

**Fig. 3:**
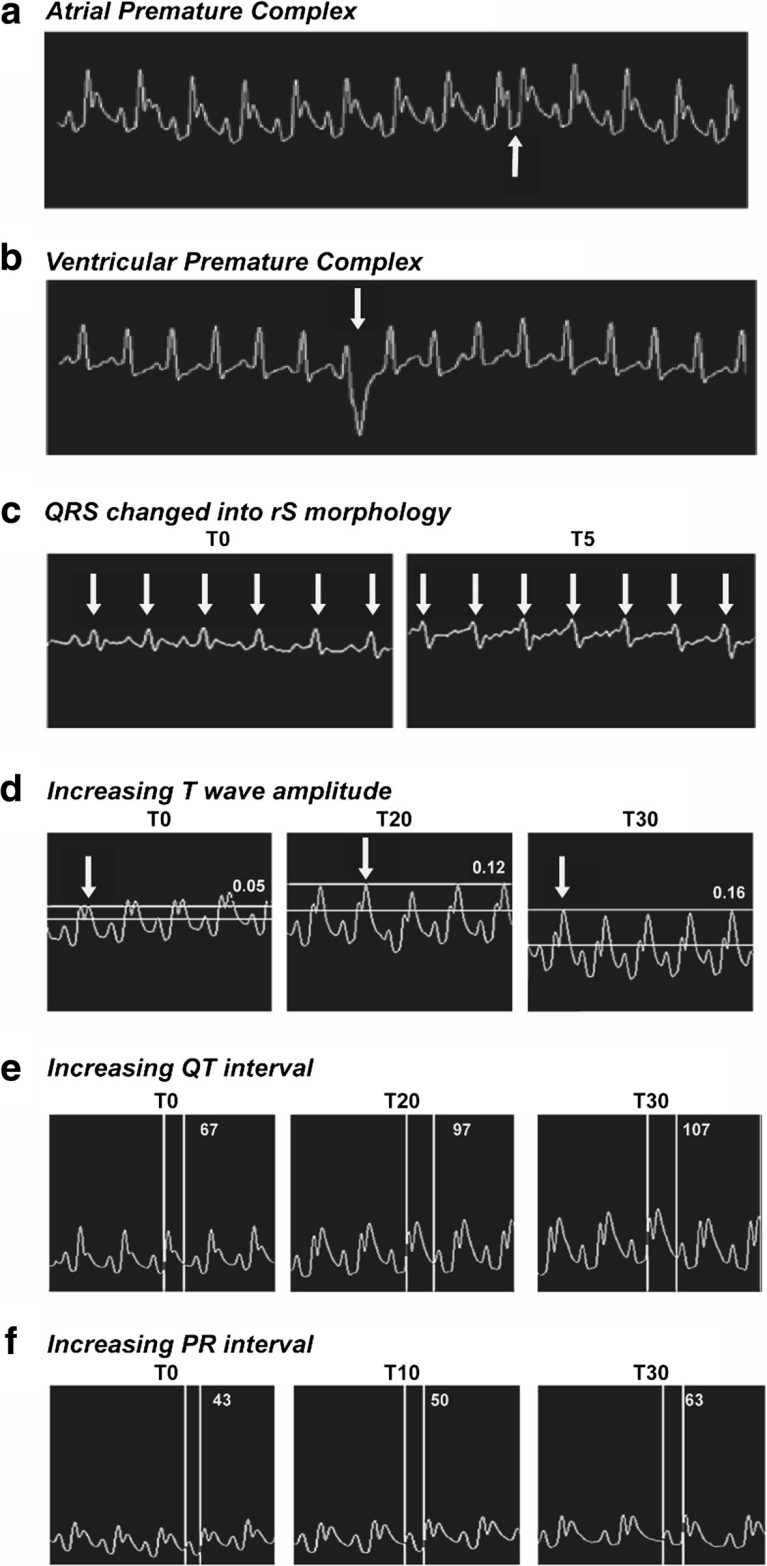
Electrocardiogram alterations in animals treated with *Hlsv*. ECG from two animals of the treated group showing (**a**) atrial premature complex and (**b**) ventricular premature complex 15 min after the envenomation. (**c**) At T0, all QRS complexes were normal. At T5, all complexes changed the morphology to rS wave. Velocity 50 mm/s, sensibility 2 N, lead II. (**d**) At T0, T wave was 0.05 mV. At T20, T wave more than doubled its amplitude, reaching 0.12 mV. At T30 T wave was 0.16 mV. (**e**) Increase on QT interval from 67 ms at T0, to 97 ms at T20 and 107 ms at T30. (**f**) Increase on PR interval from 43 ms at T0, to 50 ms at T10 and, then, to 63 ms at T30. Velocity 50 mm/s, sensibility 2 N, lead II

Arrhythmias were detected only on *Hlsv* group at T15. Alterations such as atrial premature complex (APC) (Fig. 3a) and ventricular premature complex (VPC) (Fig. 3b) were detected in two different animals.

An individual from *Hlsv* group showed rS wave at T5 (Fig. 3c). None of these alterations were observed in control group. Other sign of envenomation observed on *Hlsv* group was increased T wave amplitude. As shown in Fig. 3d, T wave increased from 0.05 mV (T0) to 0.12 mV (T20) and reached 0.16 mV (T30) (*p* < 0.05). There was no change on amplitude of T wave for control group. On the other hand, from 10 to 30 min after venom administration, T wave amplitude on *Hlsv* group was higher than control reaching the highest values at T25 and T30 (Table 1).

Alterations in QT interval (Fig. 3e) and in PR interval (Fig. 3f) in *Hlsv* group were observed. QT interval increased from 67 ms (T0) to 97 ms (T20) and 107 ms (T30) (*p* < 0.05). PR interval also increased by showing 43 ms at T0, 50 ms at T10 and 63 ms at T30 (*p* < 0.05).

### Histopathological examination

**Fig. 4:**
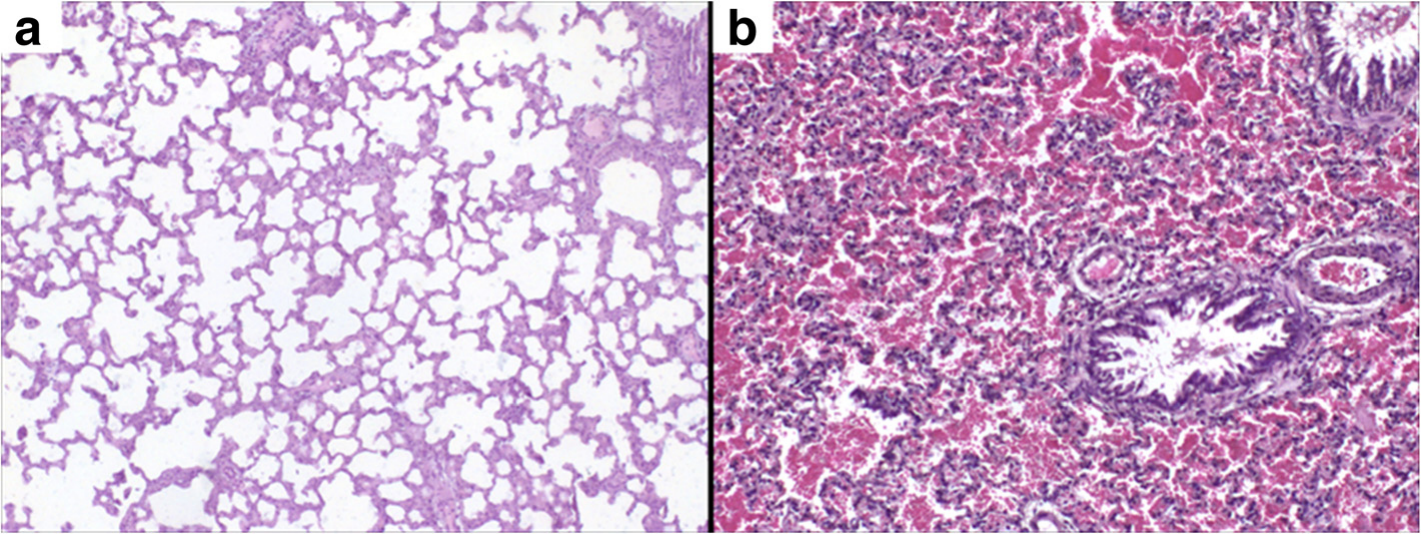
Histopathology of the lungs. (**a**) Lungs of control rats showing no alterations and (**b**) lungs with diffuse hemorrhage of animals injected with 750 μg of *H. lunatus* venom. Magnification: 40x

Lungs of animals from control and Hlsv groups were removed and analyzed microscopically. Only lungs of Hlsv treated rats showed significant alterations. Those of control animals were morphologically normal (Fig. 4a), whilst lungs of envenomed rats showed diffuse hemorrhage (Fig. 4b).

### Immunofluorescence and calcium transient

**Fig. 5:**
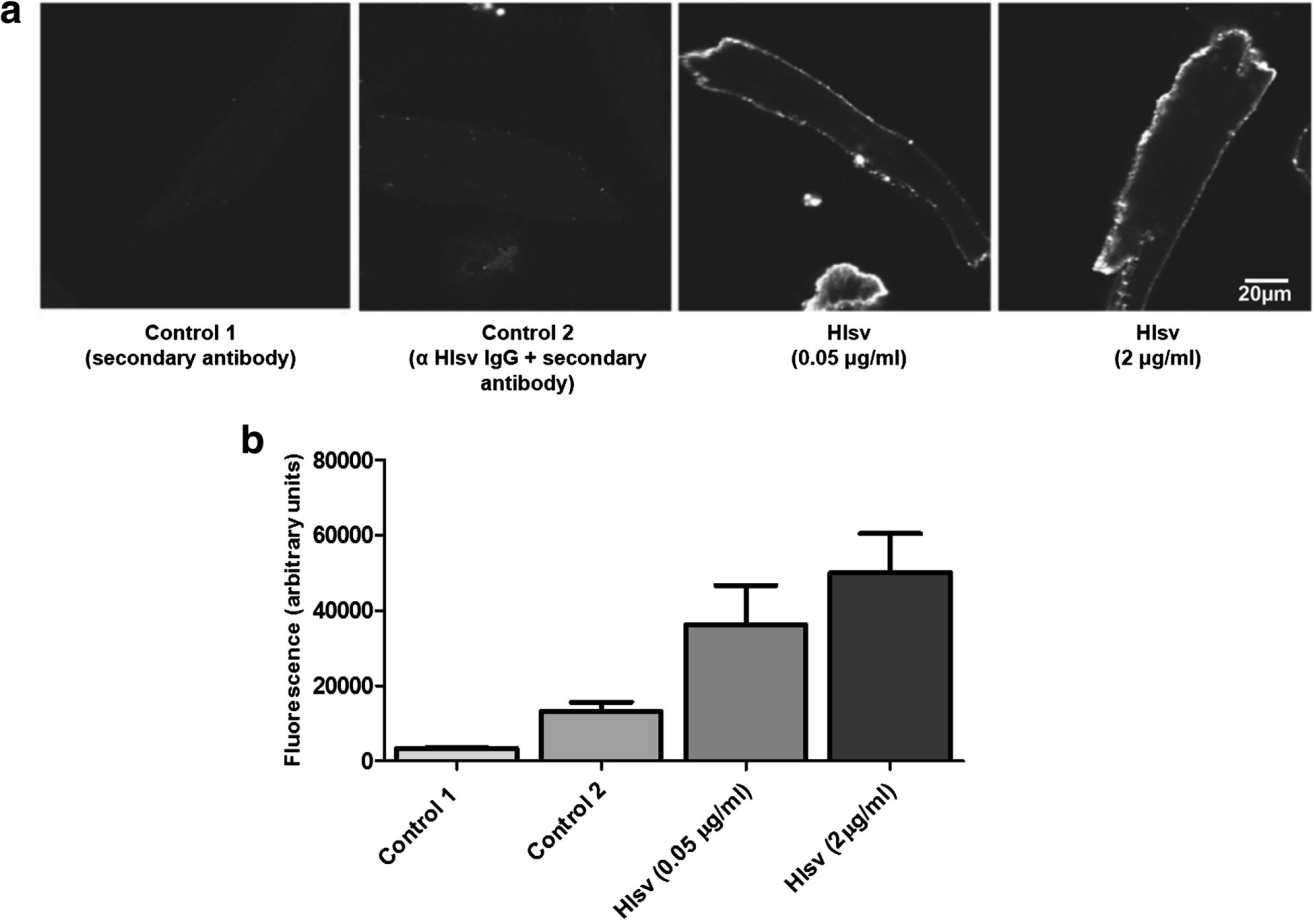
Immunolocalization of *Hlsv* in isolated mouse cardiomyocytes. **a** Localization of *Hlsv* detected with anti-*Hlsv* IgGs in ventricular myocytes. Cells were treated with *Hlsv*, incubated with anti-*Hlsv* IgGs antibodies, followed by anti-rabbit IgG conjugated to Alexa Fluor 488 (Invitrogen, USA). As control, cardiomyocytes were treated only with secondary antibodies (control 1) or with IgG anti-*Hlsv* + secondary antibodies (control 2). Seven cells were analyzed in each group. **b** Bar graph shows the concentration-dependency of venom binding to cardiac cells

To investigate whether *Hlsv* could bind to mouse ventricular cardiomyocytes, suggesting a direct cardiotoxic effect (Fig. 5), freshly isolated ventricular myocytes were treated with *Hlsv* and then incubated with anti-*Hlsv* IgG for evaluation. Fluorescent labeling was detected only in *Hlsv* treated cells (Fig. 5a). Results were analyzed using ImageJ software to quantify the fluorescence intensity (Fig. 5b). A concentration-dependent binding was observed.

**Fig. 6:**
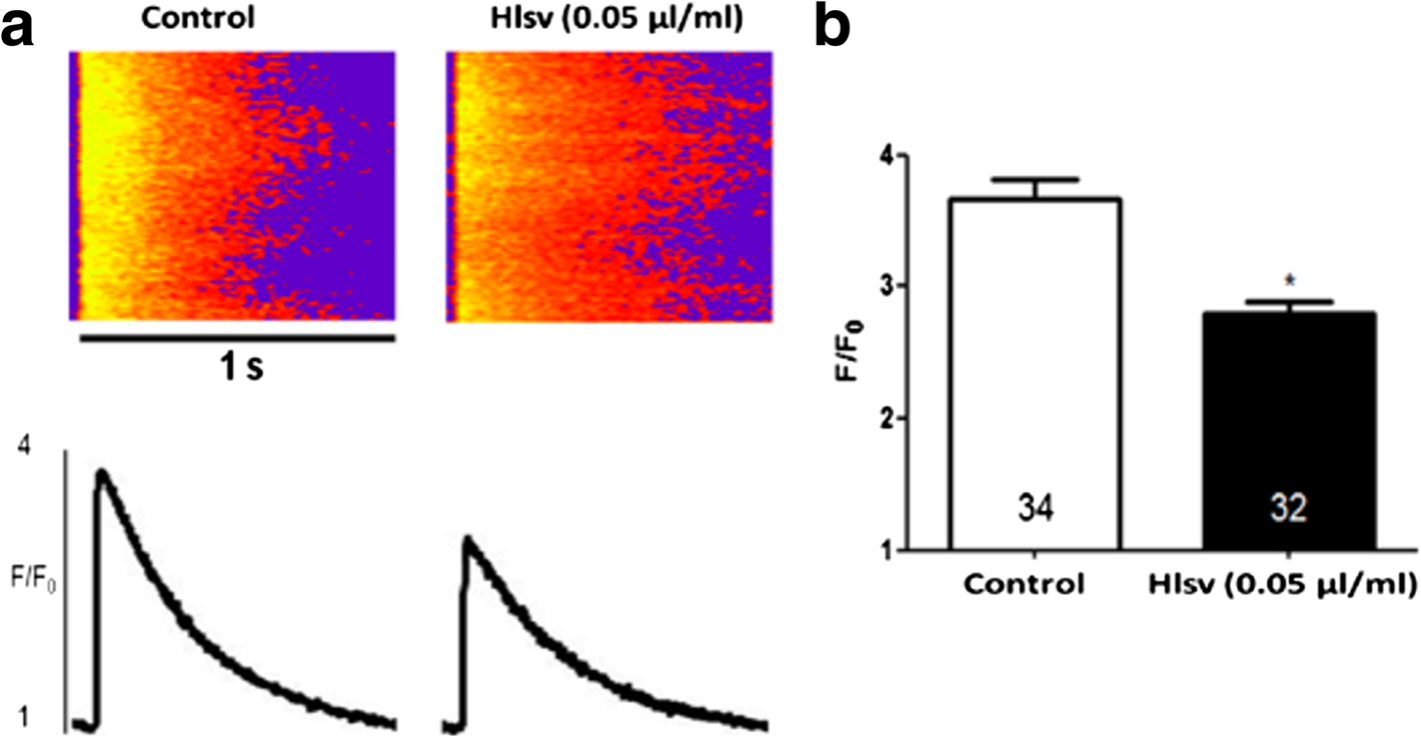
Cardiomyocytes exposed to *Hlsv* display reduced Ca^2+^ transient amplitude. **a** (*Top*) Representative confocal images of electrically stimulated intracellular Ca^2+^ transient recordings in ventricular myocytes. (*Bottom*) Ca^2+^ transient line-scan profile. **b** Significant reduction in peak Ca^2+^ transient amplitude was observed in freshly isolated adult ventricular myocytes incubated with 0.05 μg/mL of *Hlsv* venom for 5 to 20 min. Numbers inside the bars (34 in the control group and 32 in the in the treated group) represent the number of cells analyzed. Data are expressed as mean ± SEM, * *p* < 0.0001 compared to control

At last, it was investigated whether *Hlsv* could cause alterations in the calcium transient in isolated cardiomyocytes. Calcium transient amplitude, given by the ratio between maximal fluorescence (F) and baseline fluorescence (F0), was significantly reduced in cardiac cells incubated with *Hlsv* compared to control cells (Fig. 6). Thirty-four cardiomyocytes were analyzed in control group and 32 in the *Hlsv* group (Fig. 6b).

## Discussion

It is known that scorpion envenomations associated with cardiorespiratory alterations may culminate in heart failure, pulmonary edema and even death [10, 17]. The present study is the first to evaluate the consequences of *H. lunatus* venom with special attention to the cardiorespiratory system. Other than some transcriptomic analysis, little is known about *H. lunatus* and other non-Buthidae scorpion venoms and the potential harm caused by accidents with these less medically relevant scorpion species [18–21].

Since scorpion envenomation leads to adrenalin release, the increase in the heart rate detected by ECG might be a consequence of its positive chronotropic effect [22]. Venom interference on heart electric activity regulation results in ionic imbalance between intra and extracellular spaces. Consequently, the duration of cell depolarization phase is longer and leads to a hyperexcited status [23]. This explains the premature complexes of atrial or ventricular origin detected on envenomed rats.

Electrolytic imbalance has already been reported as a systemic effect of scorpion envenomation [10]. Such alteration may be diagnosed by ECG as the presence of increased T waves, which has been detected in victims of scorpionism [24]. T wave amplitude must be lower than a quarter of the R amplitude and higher values are a sensitivity parameter for electrolytic imbalance [23]. Therefore, increased T waves found in *Hlsv* group may be a consequence of electrolytic imbalance.

Moreover, rS wave detected on *Hlsv* group suggests overload of the right ventricle, due to pulmonary hemorrhage, which was confirmed by diffuse hemorrhage detected in lung histology. Occurrence of pulmonary alterations due scorpion envenomation is a common finding in the literature and pulmonary edema, hemorrhage and inflammation have already been described, but predominantly in victims of stings by Buthidae family scorpions [10, 22, 25, 26]. Describing such alterations as a result of envenomation by a species considered not very toxic is remarkable and draws attention to the fact that accidents with this scorpion should receive medical attention, since lung complications are considered the main cause of death in victims of scorpion stings [25–27].

Some of these ECG findings were also detected in a similar animal model using *Tityus fasciolatus* venom, a member of the Buthidae family, one of the most toxic scorpions species [10]. Indeed, several ECG alterations are also reported in patients severely envenomed by buthid scorpions *Tityus* [27]. *H. lunatus* does not belong to this family and yet present similar alterations, which, once more, emphasizes the importance of studying the so-called less toxic venoms and providing careful medical support to *Hlsv* envenomed victims [28].

Since cardiac alterations were detected by ECG and corroborated by prior enzymatic tests, we decided to evaluate the direct venom effect in cardiomyocytes [5]. Although the so-called “adrenergic storm”, caused by venom neurotoxin-induced discharge of catecholamines, is widely accepted as the main reason for the cardiorespiratory impairment in scorpion envenomation, it is suggested that the release of cytokines and a direct effect of venom components on the heart can also account for this clinical condition [8, 29–31]. We have previously attested the presence of neurotoxins in *Hlsv* and the augment in inflammatory cytokines following *Hlsv* administration in mice [5, 6]. In the present work, it was shown that *Hlsv* is able to directly bind to mouse cardiomyocytes. Therefore, all the possible molecular mechanisms for the onset of cardiorespiratory syndrome following scorpion envenoming seems to be present in *Hlsv.*

The decrease in calcium transient amplitude observed in cells exposed to *Hlsv* might explain some of the ECG alterations detected on treated group. It was already reported that imperatoxin I (IpTxI), a 15 kDa phospholipase from *Pandinus imperator* scorpion, induces a fast and reversible blockade of ryanodine receptors (RYR) of skeletal and heart muscles. When injected into ventricular cells, IpTxI leads to decreased amplitude of contraction and intracellular calcium transient, which indicates a blockade of calcium release from the sarcoplasmic reticulum [32]. The lipolytic fraction M1 from *Buthus occitanus tunetanus* scorpion venom was also capable of decreasing calcium transient in isolated cardiomyocytes [33]. Although the specific toxin responsible for this action was not identified, its lipolytic activity may suggest the presence of phospholipases in this fraction.

We have previously showed that *Hlsv* contains remarkable phospholipase activity [5]. The presence of a high content of proteins between 12 and 17 kDa, compatible with PLA_2_ molecular weight, was also attested by SDS-PAGE in the present work. Therefore, it is possible to suggest that an enzyme similar to IpTxI can be present in *Hlsv* and be involved in the effects observed in the present study. It has been indicated that scorpion venom PLA_2_ can also be involved in the induction of lung edema [34]. A component pertaining to this class of enzymes in *Hlsv* can be the responsible for many of the alterations described in this study. The isolation of *Hlsv* PLA_2_ would help to elucidate its role in envenomation.

## Conclusion

*H. lunatus* scorpion venom (*Hlsv*) induced cardiorespiratory alterations in experimentally envenomed rodents. The study of the pathogenesis of systemic effects provoked by this venom, and the involvement of individual venom components in the complex alterations detected, will be useful for identifying suitable therapeutic agents for treating the clinical symptoms caused by *Hlsv*.

## Abbreviations

DMEM, Dulbecco’s modified Eagle’s medium; ECG, electrocardiography; *Hlsv*, *Hadruroides lunatus* scorpion venom; HR, heart rate
